# Eruptive nevi in the setting of encorafenib: A case report and literature review

**DOI:** 10.1016/j.jdcr.2024.09.028

**Published:** 2024-11-15

**Authors:** Marisa Lenga, Samantha Venkatesh, Jennifer Nam Choi

**Affiliations:** a Department of Dermatology, Northwestern University Feinberg School of Medicine, Chicago, Illinois; b Loyola University Chicago Stritch School of Medicine, Maywood, Illinois

**Keywords:** BRAF inhibitor, encorafenib, eruptive nevi

## Introduction

Eruptive nevi is a rare phenomenon characterized by the sudden development of multiple (>10) melanocytic lesions during a distinct time frame (weeks to months), typically secondary to a disease or medication.^1^ BRAF inhibitors are one such class of medication that have been implicated in this phenomenon.

BRAF is an essential enzyme in the MAP-kinase (MAPK) signaling pathway, which regulates cellular proliferation, differentiation, survival, and angiogenesis. Mutations in the BRAF gene are implicated in the development of cancers, such as melanoma and colorectal, as these mutations lead to hyperactivation of BRAF kinase and unchecked cellular proliferation.^2^ BRAF inhibitors (BRAFi) are therapeutics that interfere with this hyperactive pathway while simultaneously promoting immune system recognition of the tumor and antitumor T cell responses.^2^ While cutaneous toxicities secondary to BRAFi, specifically vemurafenib and dabrafenib, are well documented, the cutaneous side-effect profile of encorafenib, a novel BRAFi approved for melanoma and colorectal cancers, is still largely unknown. Thus, we present a case and review the available literature on eruptive nevi secondary to encorafenib.

## Case presentation

**Fig 1:**
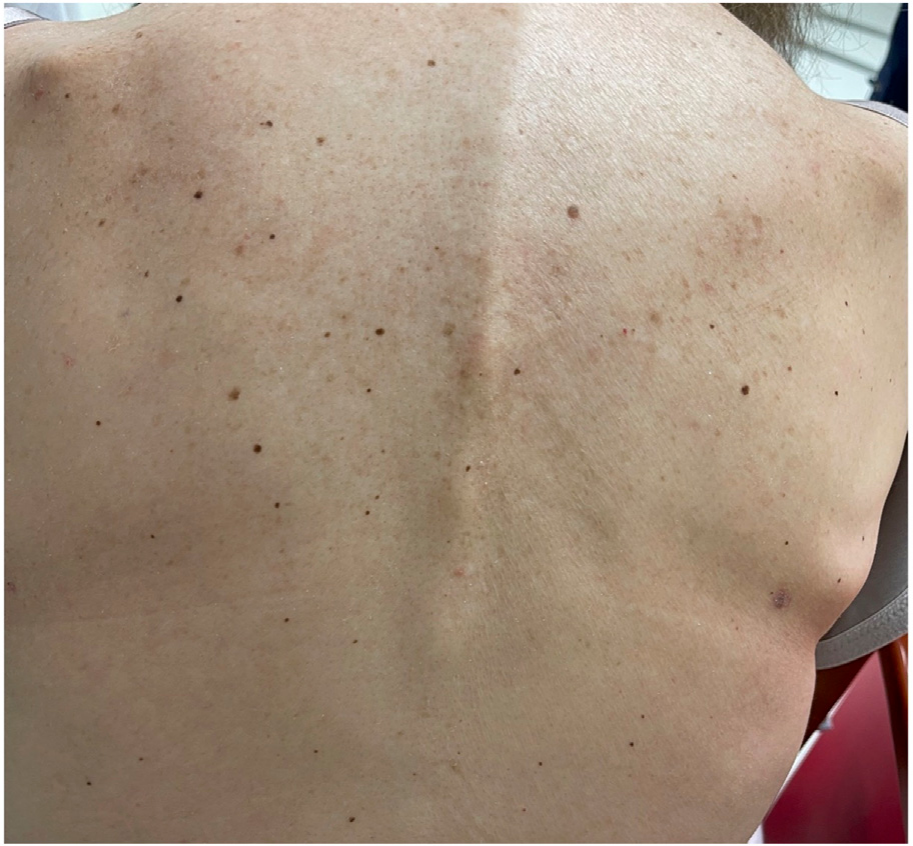
Numerous scattered 2 to 5 mm benign-appearing *brown* macules consistent with nevi on the back, bilateral upper arms, and bilateral thighs, respectively.

**Fig 2:**
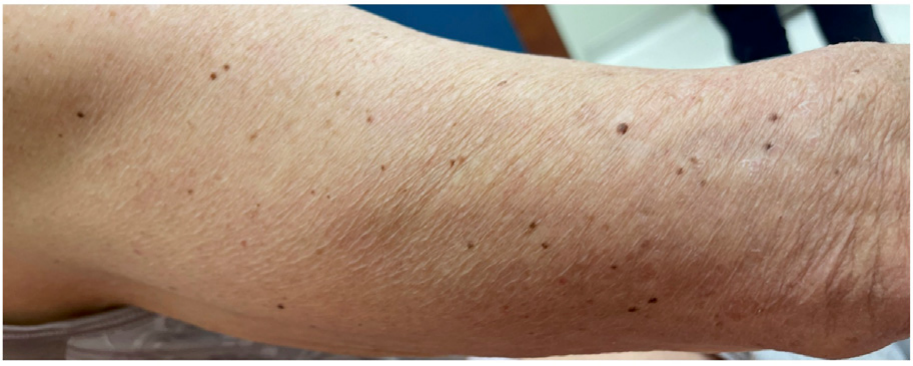
Numerous scattered 2 to 5 mm benign-appearing *brown* macules consistent with nevi on the back, bilateral upper arms, and bilateral thighs, respectively.

**Fig 3:**
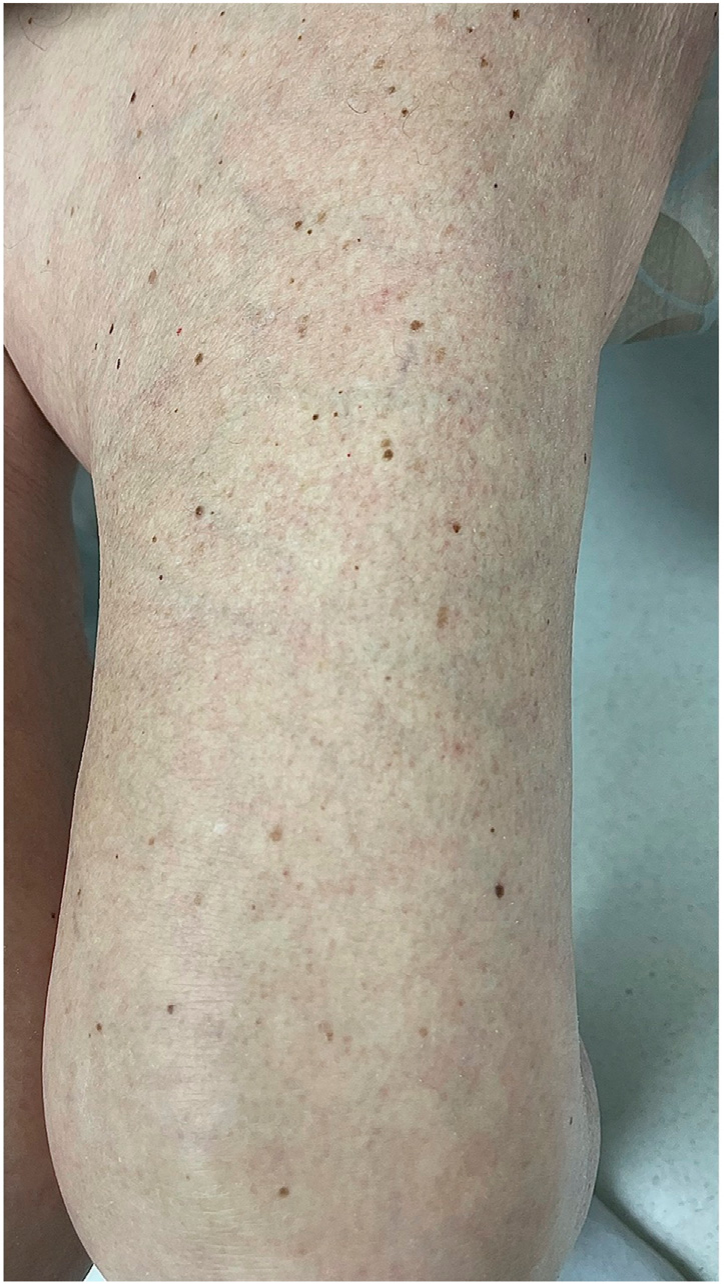
Numerous scattered 2 to 5 mm benign-appearing *brown* macules consistent with nevi on the back, bilateral upper arms, and bilateral thighs, respectively.

**Fig 4:**
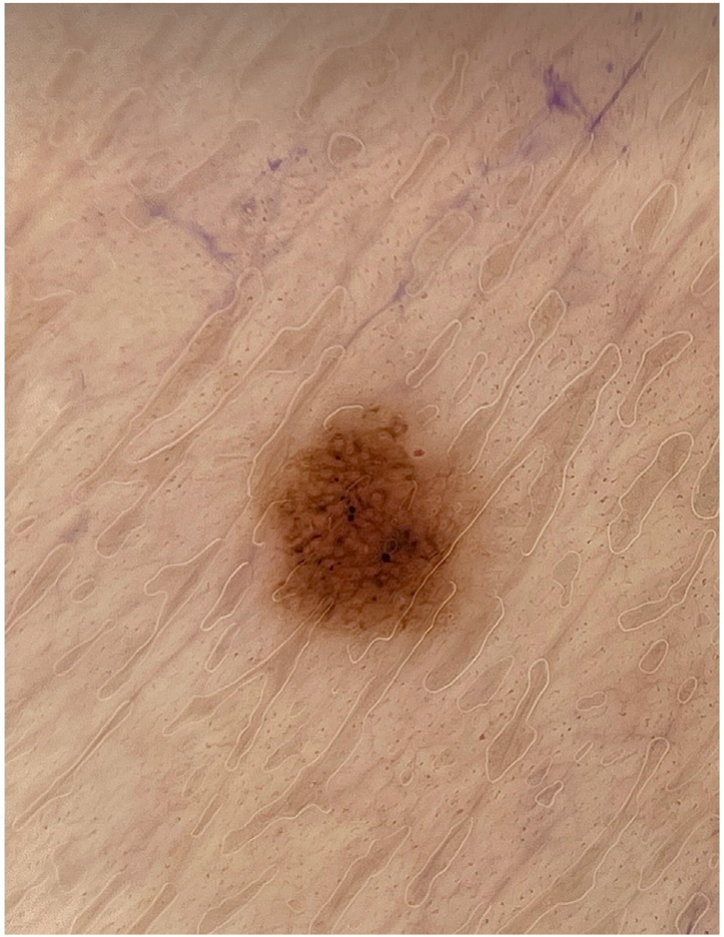
Dermoscopy revealed reticulated macules, some with scattered darker granules.

A woman in her 70s with BRAF V600E-positive colon adenocarcinoma with metastases to the lungs presented to clinic for a new, asymptomatic eruption on her arms, legs, and back that developed 8 weeks after starting encorafenib and cetuximab. This regimen was initiated after her disease progressed following cessation of FOLFOX (folinic acid, fluorouracil, and oxaliplatin) and while receiving FOLFIRI (folinic acid, fluorouracil, and irinotecan) and bevacizumab. On physical exam, the patient was Fitzpatrick Type III, and there were approximately 40 scattered, regular, 2 to 5 mm, benign-appearing nevi on the back, bilateral upper arms, and bilateral thighs (Fig 1, Fig 2, Fig 3). Dermoscopy of the nevi showed brown reticulated macules, some with scattered dark granules (Fig 4). A shave removal of one representative lesion on the left thigh was performed. Pathology demonstrated irregular nests and fascicles of melanocytes along the dermal-epidermal junction and upper dermis, cells with variable pleomorphism and hyperchromasia, and lamellar fibroplasia and scattered mononuclear infiltrate in the papillary dermis. These findings were consistent with a compound dysplastic nevus with moderate atypia. Next-generation sequencing was performed in the molecular lab and revealed a positive NRAS Q61 R variant and negative BRAF V600 E mutation. The patient was diagnosed with eruptive nevi, and no treatments for skin findings were initiated at this time. Her oncology team stopped treatment with encorafenib after 14 total weeks of treatment due to progression of disease, and the patient passed away approximately 12 weeks later.

## Discussion

Encorafenib-induced eruptive nevi is a rarely reported side effect of this therapy. Table I summarizes the demographic and clinical findings from 7 reported cases in the literature, including the present case. Of the 7 patients, 3 (42.9%) were men and 4 (57.1%) were women. All patients, if documented, were Fitzpatrick skin types II and III. The majority of patients (85.7%) were being treated for colorectal cancer, and most patients were receiving encorafenib in combination with another agent. Patients developed a range of 30 to 200 new nevi within 2 to 8 weeks following initiation of encorafenib. The most common location for new nevi to occur was on the palms, which is consistent with a previous review that found that eruptive nevi tended to develop in sun-protected sites in patients receiving targeted chemotherapies.^7^ Interestingly, our patient did not have nevi on the palms. Biopsies taken from 3 of 7 (42.8%) patients demonstrated benign compound nevi without atypia.

**Table I:** Patient demographics and clinical characteristics

Characteristic	Number of cases (*N* = 7)^1^^,^^3^, ^4^, ^5^, ^6^
Sex	
Male	3
Female	4
Age	
<50	2
50-64	3
>65	2
Fitzpatrick skin type	
II	4
III	2
Not reported	1
Cancer	
Colorectal	6
Melanoma	1
Metastases location	
Lungs	1
Liver	2
Lymph nodes	1
Not reported	4
Previous cancer treatments	
FOLFOX	3
FOLFIRI	1
FOLFOXIRI	1
FOLFIRI + Bevacizumab	1
FOLFOXIRI + Bevacizumab	3
Surgery	1
Treatment regimen that caused eruptive nevi	
Encorafenib	1
Encorafenib + Cetuximab	5
Encorafenib + Cetuximab + Binimetinib	1
Location of new nevi	
Face	3
Trunk	2
Back	2
Buttock	1
Upper extremities	4
Lower extremities	3
Palms	5
Soles	3
Not reported	1
Biopsy results	
Benign compound nevi	3
Compound nevi with mild atypia	2
Compound nevi with moderate atypia	1
Not reported	1
NGS testing	
+NRAS Q61 R	1
+NRAS Q61 K	1
Negative BRAF V600 E mutation	2
Not reported	3
Nevi follow-up	
Stable, no change in number, size, or dermoscopy	3
Spontaneous regression	1
No follow-up	3
Cessation of encorafenib	
Yes	4
No	2
Not reported	1
Mortality	
Alive	3
Dead	3
Not reported	1

The mechanism by which encorafenib induces eruptive nevi is uncertain. However, it is postulated that BRAF inhibitors binding to wild-type RAF stimulate downstream dimerization and cause a paradoxical activation of the MAPK signaling pathway in wild-type cells.^3^ This activation promotes the development of new nevi and the growth of preexisting nevi. In fact, in 4 cases of encorafenib-induced eruptive nevi where biopsies were performed, next-generational sequencing demonstrated either a positive NRAS variant or a negative BRAF V600 E mutation, suggesting upregulation of BRAF wild-type cells. While most patients were treated with both encorafenib and cetuximab, encorafenib is favored to be the inciting agent due to its mechanism on the MAPK pathway. In contrast, cetuximab inhibits epidermal growth factor receptor, leading to complete downstream inhibition the MAPK pathway.^4^ Moreover, there are currently no reports of eruptive nevi secondary to cetuximab monotherapy.

The risk of encorafenib-induced eruptive nevi developing atypia or primary melanoma is unclear. Of the 4 patients who received documented follow-up care, 3 of 4 (75%) patients’ nevi were unchanged in number, size, and color, and one patient had spontaneous regression of nevi following discontinuation of encorafenib. This reported stability is seen in other cases of eruptive nevi with BRAF inhibitors, in which most cases had an initial dynamic growth pattern and then became clinically stable over time regardless of continued treatment, treatment discontinuation, dosage reduction, or replacement with another therapy.^7^ However, the development of dysplastic nevi and new primary melanoma within pre-existing moles in patients treated with other BRAF inhibitor monotherapy has been rarely reported.^8^^,^^9^ Further research is necessary to better understand the mechanism of this phenomenon and its malignant potential.

Evidence-based management and follow-up practices for encorafenib-induced eruptive nevi are largely unknown. While most eruptive nevi in the literature remained stable, clinical surveillance of melanocytic lesions is necessary to assess the long-term malignant potential of eruptive nevi. Regular follow-up throughout the course of encorafenib treatment, potentially in pigmented lesion clinics, may be helpful to monitor old nevi and assess any new nevi.^4^ Additionally, there may be a role for the concomitant use of MEK inhibitors, as regression of eruptive nevi has been reported in patients treated with this combination.^10^ While biopsies should be performed for any changing lesions or lesions demonstrating concerning findings on dermoscopy, whole-body photography and serial dermoscopy may help monitor nevi and limit unnecessary excisions.

## Conclusion

Encorafenib is a newer BRAF inhibitor that has been implicated in the development of eruptive nevi. While majority of nevi in the reported literature were benign and stable, the risk of developing atypical nevi or melanoma is unknown. As such, clinical surveillance of nevi is essential to monitor for malignancy.

## Conflicts of interest

None disclosed.
